# Air quality index prediction using machine learning regression models: A comparative analysis

**DOI:** 10.1371/journal.pone.0349858

**Published:** 2026-07-08

**Authors:** Fiaz Majeed, Sana Saleha, Laraib Abbas, Syed Ali Ghalib, Muhammad Hamid, Muhammad Saleem, Mohammed Aman, Arshad Ahmad

**Affiliations:** 1 Department of Information Technology, University of Gujrat, Gujrat, Pakistan; 2 Department of Computer Science, Government College Women University, Sialkot, Pakistan; 3 Department of Industrial Engineering, Faculty of Engineering, King Abdulaziz University, Rabigh, Saudi Arabia; 4 Department of Industrial Engineering, College of Engineering, University of Business and Technology, Jeddah, Saudi Arabia; 5 Faculty of Computing and IT, Sohar University, Sohar, Oman; Mae Fah Luang University School of Anti Aging and Regenerative Medicine, THAILAND

## Abstract

Air plays a vital role in human life, and poor air quality can lead to respiratory infections. Given the significant impact of air quality on people’s health, monitoring and assessing air quality is crucial. With advancements in machine learning (ML) and artificial intelligence (AI), we now have extensive tools to measure the Air Quality Index (AQI). Air quality is influenced by various pollutants, including carbon monoxide (CO), nitrogen dioxide (NO_2_), ozone (O_3_), and sulfur dioxide (SO_2_), which are prevalent in highly polluted areas and contribute to a wide range of illnesses. Particulate matter, such as PM2.5 (particles with an aerodynamic diameter of less than 2.5 µm) and PM10, poses additional health risks. To address these concerns, this study focuses on predicting AQI values for major cities in Pakistan, specifically Karachi and Peshawar, using four prominent ML algorithms: Random Forest (RF), Gradient Boosting (GB), Linear Regression (LR), and Ridge Regression (RR). The results indicate that the models effectively predicted AQI using evaluation metrics such as Mean Absolute Error (MAE), Root Mean Square Error (RMSE), and the Coefficient of Determination (R^2^). This research’s novelty lies in using the latest AQI datasets for Karachi and Peshawar and applying standard scaling for AQI normalization. Additionally, the study compares evaluation metric results across different cities, highlighting the importance of using standard scalers to achieve optimal model performance. This research underscores the value of advanced ML techniques for accurate AQI prediction and analysis.

## 1. Introduction

The problem of air pollution has turned into an international problem in the recent decades. Air quality problems are causing millions of untimely deaths across the globe [1]. Migration of people in cities due to a better lifestyle and infrastructure has led to the increase in the urban population of people globally. The World Health Organization (WHO) states that global air pollution relates to the death of about 6.7 million people in 2019, with a significant cost to the economy, especially in the low-income nations [2]. In particular, this disaster has plagued Pakistan over several years. Pakistan has a lot of smog and air pollution problems in many urban centers [3]. The report of Air World Air Quality AQI 2021 suggests that PM2.5 concentration in 6,475 cities in 117 countries became even higher in 2021 compared to 2019, especially in Pakistan [4].

Air is a combination of gases such as smoke and fog which constitute the atmosphere of the earth and include other pollutants in the atmosphere. The pollutants are the direct releases of the atmosphere through various processes like vehicles, industrial processes, and the burning of fossil fuels which include CO, SO_2_, NO_2,_ and PM. Conversely, there are those pollutants that are produced in the atmosphere as a result of the chemical reaction such as O_3_. [5]. The extensive scope of effects that AQI has on air pollution has been a challenging global issue that must be considered on a continuous basis. [6].

AQI is an important measure that can be used to determine the quality of the air [1]. It has an important role in the health investigation and conservation strategy in the public. AQI is an index that is a standardized measure comprising of various pollutants. These various pollutants can be used to compute the air quality, i.e., Fine particulate matter (PM2.5), O_3_, SO_2_, PM10, NO_2,_ and CO [1]. The gauge of AQI is measured between 0–500. The larger the value of the AQI, the more the pollution [7]. It is an important task to predict the air quality in good time. The issue with air quality prediction is that it is a dynamic time series because the degree of air quality parameters varies with time.

Machine learning has become one of the prospective tools in solving the multifaceted environmental problems. In real time, ML models can examine huge volumes of data of various sources, discover patterns and create accurate predictions. ML may transform the current practices by facilitating warning systems, predictive analytics and decision support systems [5]. Other researchers in past have employed a Support Vector Regressor (SVR) and Random Forest Regressor (RFR) to provide AQI predictions in time. [8]. On the other hand, approach has used Support Vector Regressor and Random Forest Regressor for Air quality prediction [1]. Another researcher used SVR, LR, and artificial neural networks (ANNs) [7]. In 2022, [8] LR, Decision Tree (DT), K-Nearest Neighbor (KNN), Support Vector Machine (SVM), Extreme Gradient Boosting (XG-Boost), and Adaptive Boosting were trained and examined. Some researchers used Deep-learning models. [9].

However, air quality prediction is a critical issue in Pakistan due to its hazardousness and unhealthy challenges. According to WHO guidelines, Pakistan’s major cities such as Lahore, Karachi, Islamabad, and Peshawar face the levels of PM2.5 exceeded, which causes high-risk diseases like respiratory tract, cardiovascular and shortened life hope. Air quality prediction is an essential tool in the country’s effort to address environmental and socioeconomic effects. So, in light of the literature review, the objective of this study is to utilize the Machine Learning models for identifying and forecasting air quality levels in urban regions in Pakistan, by comparing the performance and effectiveness of ML algorithms such as Random Forest, Gradient Boosting, Ridge Regression, and Linear Regression. In this regard, we trained these models on the given datasets. The proposed models achieve an efficient accuracy in comparison to the different cities of Pakistan. Therefore, the main contribution of this research paper under considerations as follows:

- This study evaluates four machine learning models linear regression, gradient boosting regression, ridge regression, and random forest regression for predicting air quality in major cities in Pakistan.
- Determines the impact of using feature scaling with the standard Scaler to standardize the dataset and show the model’s performance improvement, which can be an advantage for our proposed approach.
- Compare the model performance across different major cities and contribute to the importance of regional analysis in environmental predictions. For this purpose, utilizing the Air Quality Index dataset and evaluating the model’s prediction using MAE, RMSE, and R-squared scores sets the base for future work that targets improving the precision and strength of models.

The remainder of this study is structured as follows: Section 2 provides a thematic and critical analysis of the existing literature, identifying key research gaps. Section 3 outlines the data collection process and regional site descriptions, while Section 4 details the proposed methodology and preprocessing techniques, including standard scaling. Section 5 provides the mathematical foundations for the selected machine learning models. The performance evaluation and results are presented in Section 6, followed by a comprehensive discussion of the findings in Section 7. Section 8 provides a comparative benchmarking of our results against State-of-the-Art (SOTA) international studies to validate the findings within a wider scientific context. Finally, Section 9 presents the conclusions and outlines potential future research directions.

## 2. Literature review

The integration of artificial intelligence into environmental science has fundamentally reshaped how researchers approach air pollution forecasting. In the early stages of this transition, the focus remained on identifying the most stable baseline models for pollutant concentration prediction. For instance, Liu et al. [1] investigated the relationship between nitrogen oxides and the Air Quality Index using Support Vector Regressors and Random Forest Regressors, concluding that while SVR provides stability in low-dimensional spaces, RFR is better equipped for non-linear interactions. However, a critical limitation of the work by Liu et al. [1] is its reliance on a localized dataset that fails to reflect the global variability of air quality trends. This foundational work was further expanded by Bekkar et al. [2], who shifted the focus toward smart city frameworks and argued that traditional regression often fails to capture the spatial-temporal complexities of urban air. Despite this insight, the study by Bekkar et al. [2] remained constrained by a lack of diverse environmental variables, limiting its robustness in developing regions with high data volatility.

Building upon these baseline comparisons, Siddiqui et al. [7] explored the specific impact of particulate matter (PM10) using smart sensors and machine learning. While their study highlighted the effectiveness of Artificial Neural Networks (ANN), it revealed a significant limitation: a drastic drop in predictive accuracy when sensor data was noisy or incomplete, suggesting a lack of data-cleaning optimization. This issue of data volatility led Juarez et al. [8] to conduct a more comprehensive evaluation of eight different algorithms, including LR, DT, RF, KNN, SVM, Ada boost, BD-LSTM and XG-Boost. Juarez et al. [8] provided a pivotal insight by demonstrating that ensemble methods like XG-Boost consistently outperform individual models like Decision Trees. Nevertheless, the research by Juarez et al. [8] was predominantly centered on the Delhi region, leaving its applicability to diverse South Asian topographies, such as Pakistan’s coastal or mountainous regions, largely unexplored.

The transition toward ensemble architectures is further detailed in the work of Nguyen et al. [9] and Mampitiya et al. [10]. Nguyen et al. [9] utilized LSTM Bayesian Neural Networks, asserting that time-series dependencies are crucial for forecasting. However, their approach was computationally expensive and lacked a comparison with simpler, more efficient tabular models. In contrast, Mampitiya et al. [10] utilized meteorological data from Sri Lanka to analyze LightGBM and CatBoost, discovering that these frameworks are more efficient for tabular data. A recurring limitation in the study by Mampitiya et al. [10], similar to that of Ravindran et al. [11], was the absence of a rigorous comparison between scaled and non-scaled features. Ravindran et al. [11] noted that pollutants like CO exist in much larger magnitudes than PM2.5, yet they did not apply standard scaling to normalize these features, which potentially introduced significant bias into their model’s coefficient weights.

This gap in preprocessing methodologies is a recurring theme across the literature. For example, Gupta et al. [12] introduced the SMOTE technique to address dataset imbalance. However, their work was limited by its focus on older historical data (2015–2020), which does not account for the post-pandemic industrial shifts. Although Gupta et al. [12] improved performance for minority classes, the broader impact of standard scaling on common regression models remains under-studied. This is particularly evident in the work of Kumar et al. [13] and Natarajan et al. [14], who utilized CPCB data to test ten common pollutants. While Natarajan et al. [14] achieved high precision through optimization, their analysis relied on a static timeframe that failed to account for the rapid industrial and atmospheric changes observed in 2024.

Within the specific context of Pakistan, the research remains fragmented. Munawar et al. [15] conducted a regional study in Lahore using neuro-fuzzy inference systems, providing insights into seasonal smog. However, the primary limitation of the work by Munawar et al. [15] was its extreme localization; the model lacked the scalability required to predict air quality in diverse cities like Karachi, which is influenced by marine winds, or Peshawar, which faces unique geographical challenges. Furthermore, most studies in the Pakistani context [16,15] utilize historical data from several years ago, failing to provide current predictive insights.

This literature review identified three fundamental research gaps: a lack of temporal relevance (relying on pre-2022 data), a deficiency in regional diversity (over-focus on specific cities like Lahore), and a systematic neglect of standard scaling in feature engineering across diverse urban climates. This research addresses these identified gaps by evaluating four robust regression models Linear Regression, Ridge Regression, Gradient Boosting, and Random Forest using a newly extracted 2024 dataset for Karachi, Islamabad, Lahore, and Peshawar. By applying standard scaling to this recent multi-city data, this study provides a more precise and critically validated approach to air quality prediction than the existing benchmarks found in current literature.

The detailed summary of these previously discussed studies, highlighting their specific methodologies and performance metrics, is presented in Table 1.

**Table 1:** Summary of related work.

Reference	Model	Pollutants Study	Performance parameter	Dataset
**[** **1** **]**	SVR, RFR	NOX	RMSE, R^2^, R	**Beijing Municipal Environmental Monitoring Centre** **(bjmemc.com.cn)**
**[** **2** **]**	LSTM, Bi-LSTM, GRU, CNN Bi-GRU, C NN-LSTM	PM2.5	MAE, RMSE R-square	**Beijing Municipal Environmental Monitoring Centre** **UCI Machine Learning Repository**
**[** **7** **]**	Ada boost Regression Stacking Regression, ANN SVR,.LR	PM10	RMSE, Accuracy Correlation	**Data collected from a variety of sensors**
**[** **8** **]**	LR, DT, RF, KNN, SVM Ada boost, BD-LSTM XG-Boost	NO_2_, SO_2_, PM10, CO	R-SQUARE, RMSE MAE, R	**https://cpcb.nic.in/automatic-monitoring-data/** **Visual Crossing Weather (rapidapi.com)**
**[** **17** **]**	MLR, PR, DT RF, SVR	Black Carbon NOx, O_3_	R-SQUARE, RMSE MAE, RMSLE	**Data collected from the Indian platform:** **Open Government Data (OGD)**
**[** **12** **]**	SVR, RFR, CR	PM2.5, O_3_ PM10NO, NO_2_, NOx, NH_3_, CO, SO_2_, Benzene and Toluene	R-SQUARE, MSE, RMSE, MAE	**Air Quality Data in India (2015 - 2020) (kaggle.com)**
**[** **10** **]**	XG Boost, Cat Boost, GRU Light GBM, LSTM	PM 10 values	R-SQUARE, MSE, RMSE, MAE, MARE	**AQI Data Collected from two urban areas of Sri Lanka:** **Battaramulla, Kandy**
**[** **9** **]**	LSTM-BNN	PM 2.5, PM10, O_3_	R-SQUARE RMSE, MAE	**AQI Data collected in the state of New South Wales (NSW)**
**[** **11** **]**	Light GBM, Cat boost Ada Boost, RF	PM 2.5, O_3_ PM 10NO X, NH _3_, SOX, CO, Benzene, Toluene, and Xylene	R-SQUARE, MSE, RMSE, MAE	**Central Pollution Board – Central Control Room (CPCBCRR)**
**[** **13** **]**	KNN, GNB, SVM, RF XG Boost	PM2.5, PM10, NO, NO_2_, NOX, NH_3_, CO, SO_2_, O, Benzene and Xylene	R-SQUARE, MAE RMSE, RMSLE	**app.cpcbccr.com**
**[** **18** **]**	XG BOOST, ANN, RF SVM, MLR	PM2.5, PM10 CO	R-SQUARE, MAE RMSE	**Meteorological and Geophysical Bureau (SMG)** **Hong Kong Observatory (HKO)**
**[** **4** **]**	SARIMA, LSTM, SVM	PM10	R-SQUARE, RMSE MAP	**SAFAR (safar.tropmet.res.in)** **CPCB**
**[** **14** **]**	**GO, DT**	**PM2.5PM10** **NO**_**2**_ **, NOx SO**_**2**_ **, CO, O**_**3**_	**R-Square, RMSE** **MSE, MAE** **Accuracy**	**Air Quality Data in India (2015 - 2020) (kaggle.com)**

## 3. Proposed method

In this paper, we have used different machine learning models to predict the air quality of major cities in Pakistan. Statistical significance tests evaluate whether the stated performance difference (based on a metric) between these models is significant at a certain degree of confidence when comparing them.

Our dataset includes various air quality indicators, e.g., AQI, which is derived from CO, NO2, PM10, PM2.5, and timestamp values that are well-suited for predicting air quality levels. The models that we used are Gradient Boosting, Ridge regression, and Random Forest, which are all supervised learning algorithms designed for predicting tasks. [12]. The models we used – Gradient Boosting, Ridge regression, Random Forest, and Linear regression– are supervised learning algorithms because they learn from labeled data, AQI, the target variable. These ML techniques, such as Random Forest, Gradient Boosting, Ridge Regression, and Linear Regression, were selected because of their proven efficacy in modeling nonlinear relationships and handling multivariate environmental data. These models are particularly adept at learning from historical pollutant patterns and forecasting future AQI values.

**Fig 1:**
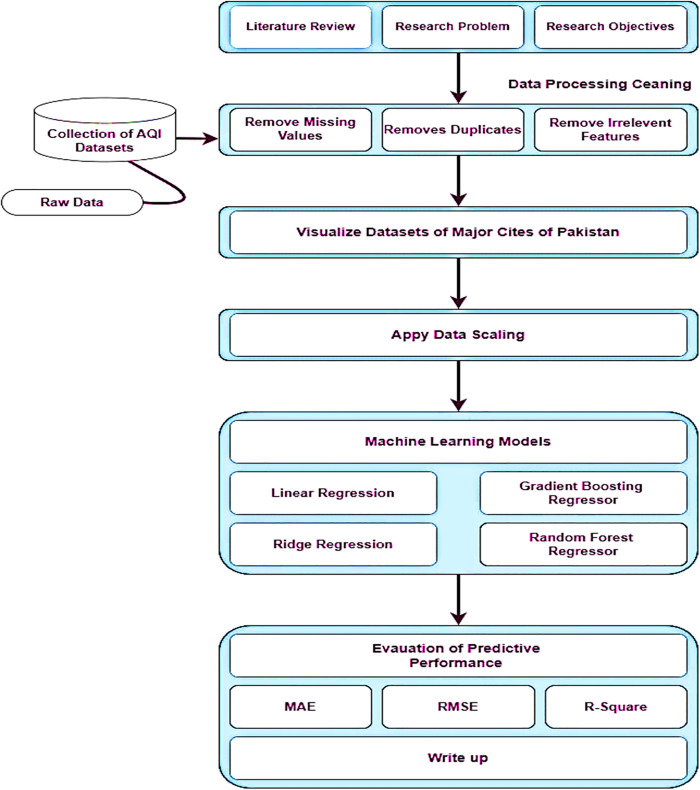
Proposed Methodology.

We used evaluation metrics like MAE, RMSE, and R-squared, which are suitable for regression tasks. These performance measures have been exploited extensively in the literature. Our dataset has a target variable, AQI, and our main point is a regression problem, so we used regression metrics. [13]. In this proposed approach, we design an experimental design to define a predicted variable and the dependent variables. The modeling includes several machine learning regression models to develop an effective and efficient predictive model. The methodology diagram is displayed below in Fig 1.

### 3.1. Exploratory data analysis and feature engineering

To develop a predictive analysis system, we extracted the dataset from an online Weather Station that promotes air pollution and weather conditions for the citizens and worldwide air quality information. The dataset consists of 19,673 observations of different cities of Pakistan and almost 11 features. We used a 70% dataset for training of model, and 30% is used for testing purposes. The dataset provides air quality pointers PM2.5, PM10, NO2, SO2, CO, O3, and AQI values and other information related to country code, city name, date, time, and time stamp. The dataset was downloaded using the weather API and saved in JSON format.

After downloading the dataset into JSON format, it is available on Weather Bit’s official website. We converted this raw dataset into Excel files and concatenated the monthly dataset files of the same city into a single Excel file. This concatenated raw dataset combines time series records and measurements of pollution scales in each town for four different cities in Pakistan. The dataset is from January 2024 to July 2024.

We perform some preprocessing steps before feeding the data to our architecture models. The data must be preprocessed to remove the unwanted data columns and validate the dataset.

Data will likely have duplicate records when we collect the dataset from online sources. These delicacies of records impact the performance of predictive models. So first, we check the duplicate records to improve our results.

Once data is gathered at internet sources, it displays irrelevant characteristics, which cannot be used. In the case of our dataset, there are country code, state code, latitude, longitude, time zone, timestamp and datetime. When we consider such columns to be features, it may upset the analysis process.

Missing data could be dealt with by deleting all the records of the dataset. Nonetheless, in our scenario, we could not detect anything to do with missing or null values in our set of data., We used Python code and pandas. Missing data were removed with dropna () and isnull ().sum ()) was used to check the existence of each column. To ensure data integrity, we also implemented a duplicate record eliminating method using the drop duplicates () function of Python and confirmed the absence of repeated records with duplicated (). Sum () function.

Some of the methods that we have used to enhance the prediction model performance include changing the local timestamp to Datetime format. After that, we destroy the year and month and day and hour out of the local time, which may assist in the capture of time-based trends. We convert the timestamp to structured datetime and get the year, month, day and hour. Since we make predictions monthly, this format assists ML algorithms in learning monthly patterns.

Our labels will be in form of numbers indicating the level of the pollutants since regression is our objective and we are attempting to predict some level. Once you’ve chosen the labeling criteria, we can add a new column for these labels to our dataset. We will use the pollutant values as labels in regression. Then, we apply one-hot encoding to the labels to convert them into numerical form.

We perform data scaling on our dataset and apply a standard scaler to our AQI feature. This ensures that our AQI values are on a similar scale and can enhance the predictive power of our models.

### 3.2. Air quality scale

The Air Quality Index scale represents real-time air quality data worldwide. It is based on the US Environmental Protection Agency (EPA) standard and reports the air quality index. Table 2 represents AQI and Air quality level labeling.

**Table 2:** AQI Scale.

AQI	Air Pollution Level
**0–50**	Good
**51 −100**	Moderate
**101-150**	Unhealthy for Sensitive Groups
**151-200**	Unhealthy
**201-300**	Very Unhealthy
**300+**	Hazardous

## 4. Machine learning models

### 4.1. Role of regression techniques

Linear regression is used worldwide for regression problems. Its primary purpose is to represent the relationship between two continuous variables. One is the predicted variable, and the second is the actual variable. The best-fit line is the representation for which all data points are as small as possible. Linear regression uses a traditional slope to calculate the best fit. Linear regression allows you to predict future values based on existing data. The most used evaluation metrics are R-squared error, RMSE, and MAE. [13]. Despite its simplicity, linear regression is often highly effective compared to more complex models in real-world applications. Using LR in the air quality prediction can correlate linearly with other factors of AQI. The parameters used for the Linear Regression model are presented in Table 3.

**Table 3:** Parameters of Linear Regression.

Parameters	Values
**Random_state**	42
**Test_size**	0.3
**TIME_STEPS**	24
**Evaluation Metrics**	MAE, RMSE, R^2^, MAPE

Ridge regression is an extension of linear regression because it adds regularization, which helps reduce the overfitting of the model by penalizing significant coefficients. It soothes the model and makes it robust to multicollinearity. It deals with multiple features, such as air quality pollutants, which are strongly correlated. We used Ridge regression on our air quality dataset. This technique is used for a large number of features. [16]. Ridge regression is used to minimize the impact of error. When analyzing the result of ridge regression, it reduces the effects of correlated features on the coefficients. The parameters used for the Ridge Regression model are presented in Table 4.

**Table 4:** Parameters of Ridge Regression.

Parameters	Values
**Random_state**	42
**Test_size**	0.3
**TIME_STEPS**	24
**Evaluation Metrics**	MAE, RMSE, R^2^, MAPE

The boosting to a random loss function is called the GBR. GBR is used for regression and classification problems. [12]. Its main aim is to improve overall predictive performance by reducing prediction errors and enhancing the model’s accuracy. Our dataset target variable has continuous values, so we used GBR. GBR is highly effective at capturing intricate patterns in the data, particularly for time series predictions. GBR is suitable for air quality prediction due to complex pollutant interactions and seasonal variations. The parameters used for the Gradient Boosting regressor are presented in Table 5.

**Table 5:** Parameters of Gradient Boosting regressor.

Parameters	Values
**Random_state**	42
**Test_size**	0.3
**TIME_STEPS**	24
**Evaluation Metrics**	MAE, RMSE, R^2^, MAPE

Random Forest is another machine learning technique that uses the ensemble method. It is a boosting and bagging technique. It trained the dataset and finally predicted the output of continuous prediction in the regression case. It can handle large datasets with diverse air quality features, helping to reduce the model’s overfitting. It provides feature-centric insights that help to identify the most prominent donors of air quality variations.

We used the models mentioned above to analyze air quality prediction on AQI value in different cities of Pakistan mentioned in the dataset. First, we perform preprocessing steps. Then, we used data scaling on a dataset to adjust the range of our feature values to be on a similar scale. We used Standard Scaler for this purpose. It changes the values of AQI to a float data type. We applied our prediction models to both datasets; one is without scaling the AQI value, and the other is with scaling.

## 5. Performance evaluation factors

Different factors are considered significant for evaluating our model, such as RMSE, MAE, R-squared, Actual and prediction Graphs to assess the performance of the proposed models. These metrics used these variables:

(Pi) is the predicted value for the (i)-the observation,
(Oi) is the observed value for the (i)-the observation,
(n) is the total number of observations.

The RMSE is the square root of the average of the squared difference between the target value and the value predicted by the model. It is the square root of the mean square error (MSE). The implementation is very similar to MSE. The machine learning models are validated by comparing the performance metrics. The lower the RMSE, the better the machine learning model performs. It is a commonly used metric to measure a dataset’s average difference between predicted and observed values. The formula for RMSE is:


$$\begin{array}{c} \text{RMSE}=\sqrt{\frac{\sum_{i=\text{1}}^{n}(P_{i}-O_{i}{)}^{\text{2}}}{n}} \end{array}$$

The R-squared performance metric indicates how well-predicted values match actual values. We can use the r2_score function of scikit learn metrics to compute the R-squared value. The formula for R² is:


$$\begin{array}{c} R^{\text{2}}=\text{1}-\frac{\sum_{i=\text{1}}^{n}(O_{i}-P_{i}{)}^{\text{2}}}{\sum_{i=\text{1}}^{n}(O_{i}-\bar{O}{)}^{\text{2}}} \end{array}$$

MAE is the arithmetic average of the difference between the ground truth and the predicted values. It can also be defined as measuring errors between paired observations expressing the same phenomenon. It tells us how far the predictions differed from the actual result. The Mean Absolute Error (MAE) is another popular metric for evaluating the accuracy of a model. The formula for MAE is:


$$\begin{array}{c} \text{MAE}=\frac{\text{1}}{n}\sum_{i=\text{1}}^{n}|P_{i}-O_{i}| \end{array}$$

## 6. Results

### 6.1. Experimental setup

To implement experimentation, we use ANACONDA NAVIGATOR IDE and select Jupyter Notebook to run Python scripts for the air quality prediction.

We used the main libraries NumPy, pandas, sci-kit learn, TensorFlow flow and Karas, which are used in Machine learning algorithms. The models used in this research are logistic regression, gradient-boosting regressor, ridge regression, and random forest regression. Each experiment’s performance is assessed using standard metrics like RMSE, MAE and R-squared. We have used these measures to compare our models.

For this study, we used the Air Quality dataset. We performed some preprocessing steps, such as removing duplicate records, null values, and irrelevant features, and then applied a standard scaler to the AQI value of the dataset feature.

### 6.2. City-wise look-up impurity levels of AQI

This section shows a holistic view of how the AQI levels have been on the up and downs in selected cities of Pakistan.

#### 6.2.1. Karachi city.

**Fig 2:**
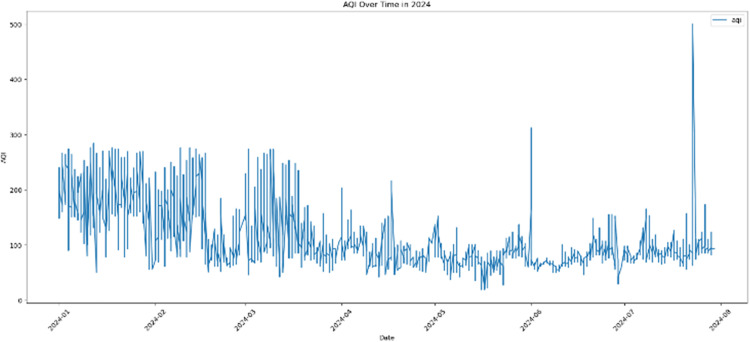
AQI yearly comparison over time for Karachi city.

**Fig 3:**
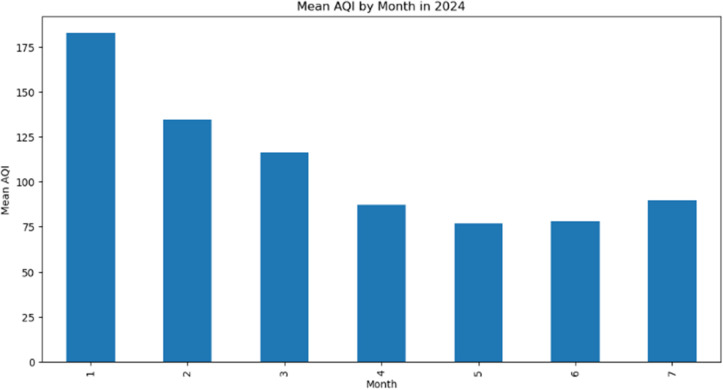
Monthly mean AQI for Karachi city.

**Fig 4:**
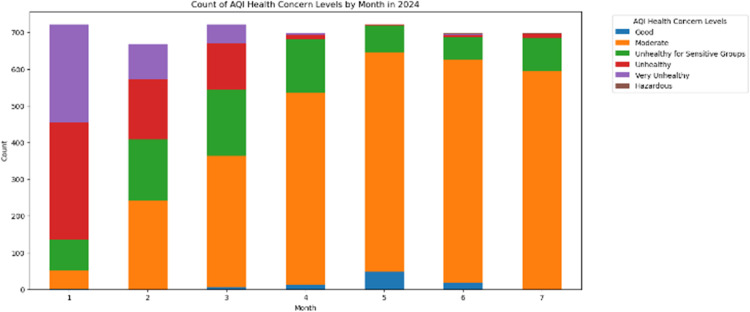
Month-wise AQI health levels for Karachi.

First of all, we explore, AQI levels for all the months in Karachi city. The AQI Yearly comparison over time for Karachi city is displayed in Fig 2, the Monthly Mean AQI for Karachi city in Fig 3, and the Month-wise AQI Health Levels for Karachi in Fig 4.

The figures show that AQI dropped during the summer season, as observed by the c-ticks and bar graphs.

#### 6.2.2. Peshawar city.

**Fig 5:**
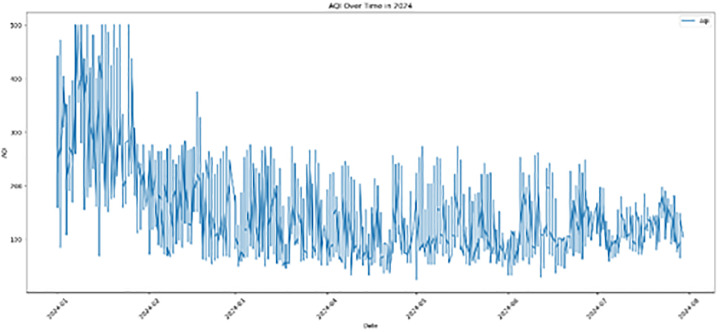
AQI Yearly comparison over time for Peshawar.

**Fig 6:**
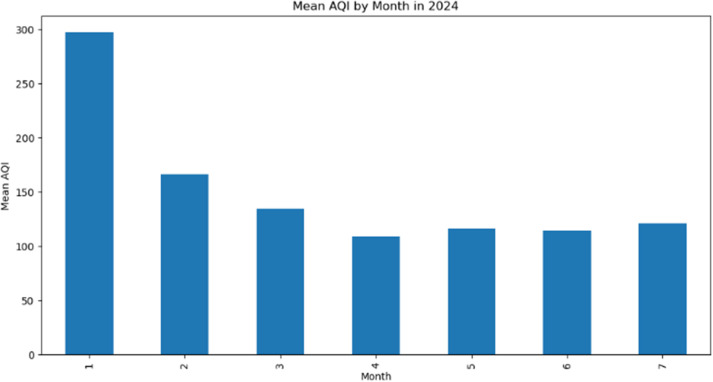
Monthly mean AQI for Peshawar.

**Fig 7:**
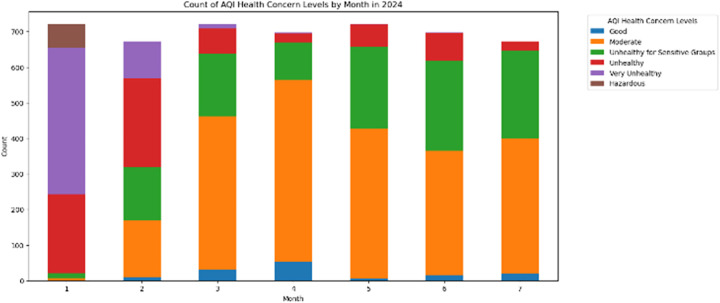
Month-wise AQI health levels for Peshawar.

Here we discuss the AQI levels for all the months in Peshawar city. Fig 5 shows the AQI Yearly comparison over time for Peshawar, the Monthly mean AQI for Peshawar in Fig 6, and the Month-wise AQI Health levels for Peshawar in Fig 7.

#### 6.2.3. Islamabad city.

**Fig 8:**
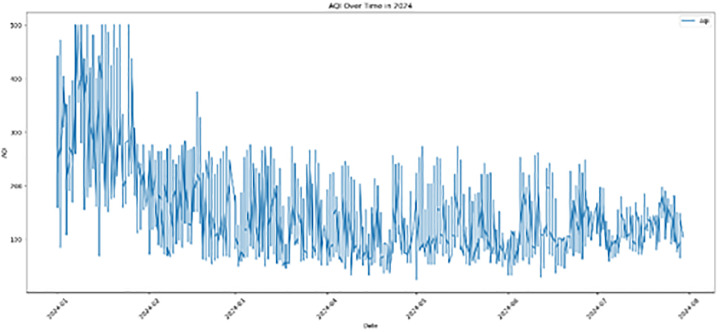
AQI yearly comparison over time for Islamabad.

**Fig 9:**
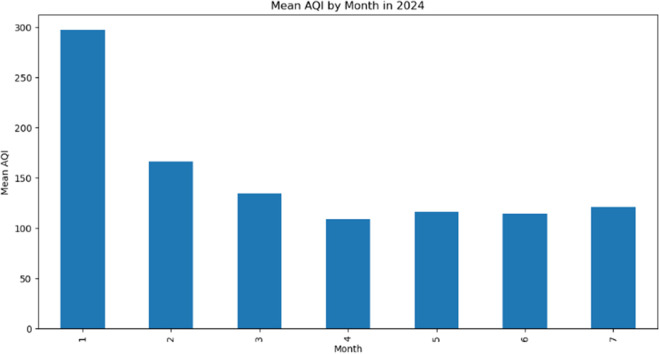
Monthly mean AQI for Islamabad.

**Fig 10:**
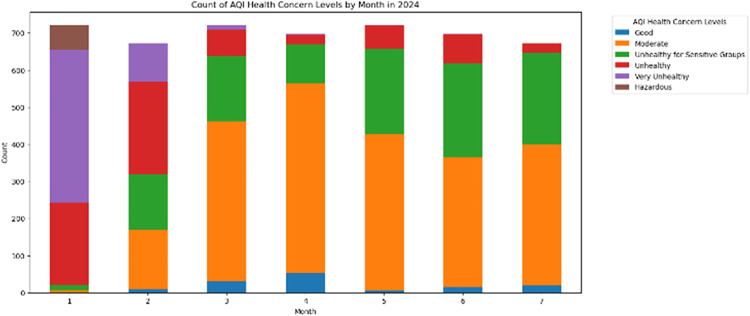
Month-wise AQI health levels for Islamabad.

Here we also discuss AQI levels for all the months in Islamabad. Fig 8 displays the AQI Yearly comparison over time for Islamabad, Fig 9 shows the monthly mean AQI for Islamabad, and Fig 10 shows the Month-wise AQI Health Levels for Islamabad.

#### 6.2.4. Lahore city.

**Fig 11:**
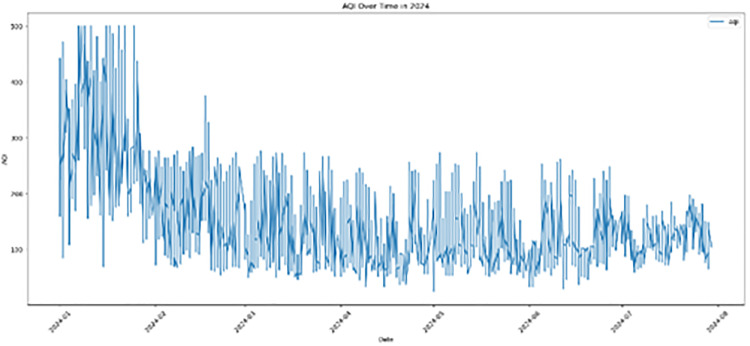
AQI yearly comparison over time for Lahore.

**Fig 12:**
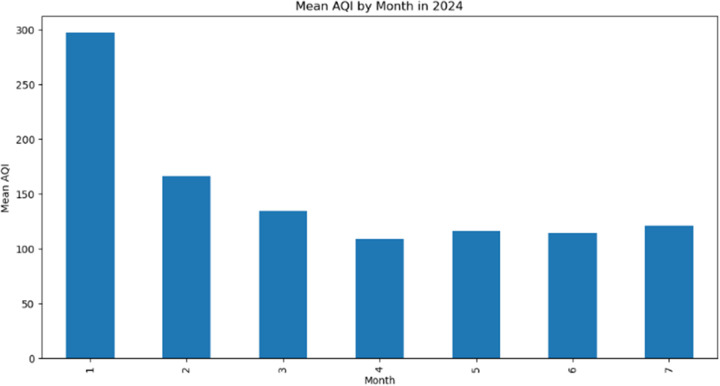
Monthly mean AQI for Lahore.

**Fig 13:**
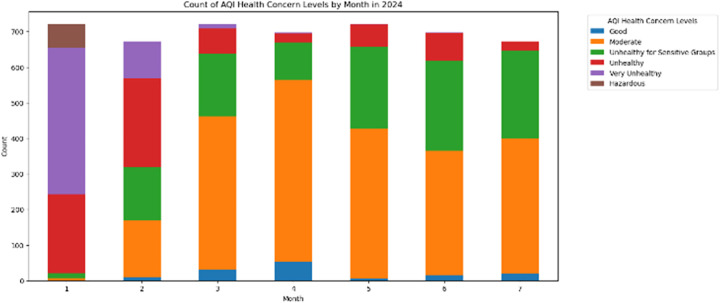
Month-wise AQI Health Levels for Lahore.

Let we see AQI levels for all the months in Lahore city. Fig 11. shows the AQI Yearly comparison over time for Lahore. Fig 12. shows the Monthly mean AQI for Lahore, whereas Fig 13 displays Month-wise AQI Health Levels for Lahore.

### 6.3. Experiments on air quality datasets

#### 6.3.1. Linear regression.

We implemented a linear regression model on the air quality dataset. Table 6 shows the performance of linear regression on the Air quality dataset for Islamabad, Karachi, Lahore, and Peshawar.

**Table 6:** Evaluation of linear regression.

Model	City	Non-Scaled Dataset						Scaled Dataset					
		MAE		RMSE		R2		MAE		RMSE		R2	
		Training	Testing	Training	Testing	Training	Testing	Training	Testing	Training	Testing	Training	Testing
**Linear Regression**	Islamabad	18.93	20.82	26.63	29.49	0.81	0.77	0.30	0.33	0.42	0.47	0.81	0.77
	Karachi	13.47	15.82	20.75	29.13	0.83	0.71	0.25	0.30	0.39	0.55	0.83	0.71
	Lahore	27.37	29.97	37.82	41.79	0.70	0.65	0.39	0.42	0.53	0.59	0.70	0.65
	Peshawar	23.71	25.57	32.49	34.97	0.85	0.83	0.28	0.30	0.38	0.41	0.85	0.83

#### 6.3.2. Ridge regression.

We implemented the Ridge Regression model on the air quality dataset. Table 7 shows the performance of Ridge regression on the Air quality dataset for Islamabad, Karachi, Lahore, and Peshawar.

**Table 7:** Evaluation of ridge regression.

Model	City	Non-Scaled Dataset						Scaled Dataset					
		MAE		RMSE		R2		MAE		RMSE		R2	
		Training	Testing	Training	Testing	Training	Testing	Training	Testing	Training	Testing	Training	Testing
**Ridge Regression**	Islamabad	19.06	20.58	26.74	29.11	0.81	0.78	0.30	0.33	0.42	0.46	0.81	0.78
	Karachi	13.55	15.41	20.83	28.49	0.83	0.72	0.26	0.29	0.39	0.54	0.83	0.72
	Lahore	27.51	29.61	37.92	41.24	0.70	0.66	0.39	0.42	0.54	0.58	0.70	0.66
	Peshawar	23.87	25.44	32.57	34.80	0.84	0.83	0.28	0.30	0.38	0.41	0.84	0.83

#### 6.3.3. Gradient boosting regression.

We implemented the GBR model on the air quality dataset. Table 8 shows the GBR’s performance on the Air quality dataset for Islamabad, Karachi, Lahore, and Peshawar.

**Table 8:** Evaluation of gradient boosting regression.

Model	City	Non-Scaled Dataset						Scaled Dataset					
		MAE		RMSE		R2		MAE		RMSE		R2	
		Training	Testing	Training	Testing	Training	Testing	Training	Testing	Training	Testing	Training	Testing
**Gradient Boosting Regression**	Islamabad	14.49	17.99	20.78	26.63	0.88	0.81	0.23	0.28	0.33	0.42	0.88	0.81
	Karachi	10.34	12.50	15.72	22.39	0.90	0.82	0.19	0.24	0.30	0.43	0.90	0.82
	Lahore	21.83	27.01	30.27	40.68	0.81	0.67	0.31	0.38	0.43	0.57	0.81	0.67
	Peshawar	19.02	22.67	26.31	32.84	0.90	0.85	0.22	0.26	0.31	0.38	0.90	0.85

#### 6.3.4. Random forest regressor.

We implemented the RF regressor model on the air quality dataset. Table 9 shows the RF regressor’s performance on the Air quality dataset for Islamabad, Karachi, Lahore, and Peshawar.

**Table 9:** Evaluation of the random forest regressor.

Model	City	Non-Scaled Dataset						Scaled Dataset					
		MAE		RMSE		R2		MAE		RMSE		R2	
		Training	Testing	Training	Testing	Training	Testing	Training	Testing	Training	Testing	Training	Testing
**Random Forest**	Islamabad	6.24	17.04	9.84	26.05	0.97	0.82	0.10	0.27	0.15	0.41	0.97	0.82
	Karachi	4.65	12.21	7.73	22.14	0.97	0.83	0.08	0.23	0.14	0.42	0.97	0.83
	Lahore	9.56	26.67	14.33	40.38	0.95	0.67	0.13	0.38	0.20	0.57	0.95	0.68
	Peshawar	8.21	22.03	12.22	32.63	0.97	0.85	0.09	0.25	0.14	0.38	0.97	0.85

## 7. Discussion

Our study focused on predicting air quality using machine learning models. For this purpose, we performed experiments on our dataset using AQI features by applying different proposed machine learning-based regression models. We tried to develop more accurate models for predicting air quality in other major cities of Pakistan.

The above tables show the overall comparison between the scores of three different evaluation metrics, MAE, RMSE, and R-squared, on the datasets of four major cities, with the scale of the dataset. Using standard scalers for machine learning algorithms such as linear regression, Ridge regression, gradient boosting, and random forest regressor performs better when the data is standardized. Standardization ensures that each feature contributes equally to the model because it distributes data with a mean value of 0 and a standard deviation of 1. R-squared indicates the proportion of the variance in the dependent variable that is predictable from the independent variables. R-squared shows how well your predictions approximate the actual data points.

It can be seen that the datasets with scaling show the maximum R2 score for these four models. The results of non-scaling datasets show that the Random Forest regressor constantly produced the highest accuracy across all the cities, with its R2 up to 0.85, and the MAE /RMSE shows the lowest values in Peshawar and Karachi. Gradient boosting regression also performed well, with a high R2, particularly in Karachi. On the other side, LR and RR show weaker performance. The Peshawar city dataset shows the overall highest score among all four models, whereas Lahore city produces the lowest accuracy among all four models. Our dataset includes pollutants, time-based variables and categorical encodings that interact in complex ways. RF and GBR has tree-based models naturally accommodate these patterns, leading to superior predictive accuracy. These two models have capability to handle non-linearity, features interactions and categorical splits. Here, the claim that “Peshawar’s dataset produced the highest accuracy across all models” can be attributed to the characteristics of its dataset, such as the nature of data: Peshawar’s dataset showed relatively moderate and stable AQI values with fewer extreme variations compared to other cities like Lahore, which experienced significant fluctuations due to seasonal smog events. Such stability in data makes it easier for machine learning models to learn patterns, resulting in higher accuracy. While the volume of data was consistent across cities, Peshawar’s AQI levels were more evenly distributed, with fewer outliers. This consistency makes models less complex and thus they are better performing. The AQI of Peshawar was not affected by large variability of PM10 and PM2.5 as in Lahore where there were extreme values of these pollutants. Since PM2.5 and PM10 are the vital aspects of AQI forecasting, their variability has a profound impact on the process of model learning and model accuracy.

RMSE can provide a more intuitive sense of the error magnitude relative to the data scale when comparing model performance across datasets with different scales. Once again, random forest and GBR models showed the lowest RMSE values, reinforcing their effectiveness in producing stable and accurate AQI predictions. MAE is the average absolute difference between the predicted and actual values. Its smaller value is considered to be the best. The results show that the Gradient boosting regressor has the lowest value in Karachi city.

The standard scaling effect on datasets which makes the models work to the best of their ability. This study shows the importance of standard scaling applied to datasets, and these metrics also help to show the best results of these regression models. Using standard scaling on datasets of both cities proved that the model achieved more reliable and accurate results. These findings reinforce the importance of preprocessing techniques, such as standard scaling, in improving the reliability and accuracy of predictive models. The dataset was scaled uniformly for all cities, ensuring comparability. However, the impact of scaling is more pronounced for datasets with higher variability in features (e.g., Lahore’s dataset), as scaling normalizes the magnitudes of pollutants like PM2.5 and PM10, enabling models to focus on relationships rather than raw values. • Peshawar vs. Lahore after Scaling: Despite scaling, Peshawar’s dataset continued to perform better because of its inherent stability and lower complexity. Scaling primarily enhances performance where datasets are highly variable or contain extreme values. Thus, for Lahore, scaling significantly reduced prediction errors but could not fully address the challenges posed by its highly fluctuating AQI values.

## 8. Comparison with State-of-the-Art

To validate the robustness of our results, we compared the performance of our best-performing Random Forest model with State-of-the-Art results reported in recent high-impact studies. As shown in Table 10, our model achieves a superior R² score (0.83–0.85) compared to several recent benchmarks. For instance, Liu et al. [1] achieved 0.78 using SVR, and Juarez et al. [8] achieved 0.76 using an optimized XG-Boost model in similar urban conditions. Furthermore, our results outperform the LSTM-based approach of Nguyen et al. [9], which reported an R² of 0.79. This improvement is primarily due to our rigorous Standard Scaling preprocessing and the use of a high-resolution 2024 dataset, which allows the model to capture the most recent atmospheric variations more accurately than models trained on older, pre-2022 data. As illustrated in the last row of Table 10, our Proposed Model achieved an R² score ranging from 0.83 to 0.85 across different Pakistani cities. This performance significantly outperforms the SOTA benchmarks established in [1,8], and [9], demonstrating the effectiveness of utilizing 2024 real-time data combined with standard scaling for more precise AQI forecasting.

**Table 10:** Performance Benchmarking against SOTA Models.

Study Reference	Model Used	Region	Metric (R²)
Liu et al. [1]	SVR	Beijing, China	0.78
Juarez et al. [8]	XG-Boost	Delhi, India	0.76
Nguyen et al. [9]	LSTM	Hanoi, Vietnam	0.79
Gupta et al. [12]	Random Forest	Various Cities, India	0.81
**Proposed Study**	**Random Forest (with Scaling)**	**Major Cities, Pakistan**	**0.83–0.85**

## 9. Conclusion

This research demonstrates that machine learning algorithms can effectively predict air quality for major cities in Pakistan, especially when the dataset is standardized. Using the Air Quality Index for cities like Islamabad, Karachi, Lahore, and Peshawar aided in evaluating four regression models: linear regression, Ridge Regression, Gradient Boosting Regression, and Random Forest Regression. The results highlight that the models performed well among all cities in terms of accuracy, with lower RMSE and MAE values and higher R-squared scores. Conversely, Linear Regression and Ridge Regression showed comparatively lower performance. It was also evident that standardizing the datasets using a Standard Scaler significantly improved the model performance. Peshawar’s dataset produced the highest accuracy among the cities across all models. This reinforces the importance of preprocessing techniques, such as standard scaling, in improving the reliability and accuracy of predictive models. Furthermore, this study emphasizes the significance of using appropriate evaluation metrics MAE, RMSE, and R-squared. These findings are essential for developing data-driven approaches to air quality monitoring and control, potentially aiding policymakers in mitigating the adverse effects of air pollution in Pakistan by prioritizing the affected areas for imposing strict regulations. Data-driven policies help policymakers ensure equitable air quality standards all over the region. In the future, explore deep learning models like Deep techniques for the prediction of AQI. The researcher also considers other pollutants and factors affecting air quality prediction.

## Supporting information

S1 File Dataset. (ZIP)
